# Practical Considerations for Next-Generation Adjuvant Development and Translation

**DOI:** 10.3390/pharmaceutics15071850

**Published:** 2023-06-29

**Authors:** William R. Lykins, Christopher B. Fox

**Affiliations:** Access to Advanced Health Institute, Seattle, WA 98102, USA

**Keywords:** adjuvants, formulation, future perspectives, vaccine development, natural product discovery, route of administration, design of experiments, systems immunology, mucosal vaccine, sustainability

## Abstract

Over the last several years, there has been increased interest from academia and the pharmaceutical/biotech industry in the development of vaccine adjuvants for new and emerging vaccine modalities. Despite this, vaccine adjuvant development still has some of the longest timelines in the pharmaceutical space, from discovery to clinical approval. The reasons for this are manyfold and range from complexities in translation from animal to human models, concerns about safety or reactogenicity, to challenges in sourcing the necessary raw materials at scale. In this review, we will describe the current state of the art for many adjuvant technologies and how they should be approached or applied in the development of new vaccine products. We postulate that there are many factors to be considered and tools to be applied earlier on in the vaccine development pipeline to improve the likelihood of clinical success. These recommendations may require a modified approach to some of the common practices in new product development but would result in more accessible and practical adjuvant-containing products.

## 1. Introduction

Throughout the last two decades, there has been increasing focus on the discovery and translation of new immune-stimulating agents [1,2]. These compounds are often collectively referred to as adjuvants due to their precedent of use in vaccine development. In recent years, there has been an expansion in the application of adjuvants in oncology and other areas as our understanding and definition of adjuvants continue to grow [3]. Adjuvants stimulate key cell types in the innate immune system and can influence the scale and class of immune response directed towards a given antigen or antigens [4]. These innate immune cell subsets can then engage with effector cells in the adaptive immune system—e.g., T and B cells—which then mediate the development of cellular and humoral immunity, respectively. Adjuvants are also critical in the development of long-term memory cell populations, which in turn can lead to years-long protection [5,6]. In existing clinical products, adjuvants are often combined with recombinant subunit antigens, which are generally not sufficiently immunogenic to generate protective immune responses on their own [7].

In recent years, there have been a handful of successful FDA approvals for vaccines containing new-to-market adjuvant systems, such as AS01, developed by GSK and included in Shingrix, Mosquirix, and Arexvy [8]. However, despite its success, AS01 is based on techniques and technologies that were developed decades ago, and many reports note that vaccine adjuvants have some of the longest times to commercialization of any clinical product [1]. A marked need exists for new adjuvant and immunostimulatory systems; however, there is also a need to better understand and plan for the barriers to translation earlier on in the adjuvant development pipeline.

The most widely used class of adjuvants is Alum, a generic term applied to aluminum salts, such as aluminum phosphate, aluminum hydroxide, aluminum hydroxyphosphate sulfate, and aluminum oxyhydroxide. Alum often serves the useful functions of adsorbing the protein antigen and mediating its presentation to innate immune cells in draining lymph nodes [4,9]. Aluminum-based adjuvants are present in many common vaccines, including the 9-valent HPV vaccine Gardasil and the Tdap vaccines (among many others) [10]. Another common class of adjuvants is oil-in-water emulsions (e.g., MF59 and AS03), which typically include squalene in the oil phase, emulsifying agent(s), and other excipients [4,11]. Much like Alum adjuvants, squalene emulsions do not appear to engage a specific pattern recognition receptor (PRR) but instead mediate a local inflammatory response and increase the trafficking of antigens to draining lymph nodes [12]. Notably, the tetravalent influenza vaccine Fluad contains MF59 [10]. Similarly, saponin-based adjuvants, such as Matrix-M, offer pleiotropic modes of action, which tend to augment humoral and cellular immune responses. Likewise, the saponin QS-21 functions through an unknown receptor(s) but is known to trigger the inflammasome in model systems of immunogenicity [13].

In contrast to the particulate adjuvant formulations described above, another class of clinical adjuvants includes specific chemical triggers of defined pathways. Most of these agents signal through PRRs, which are a critical part of the innate immune system and are involved in the detection of pathogen-associated molecular patterns (PAMPs) [14]. The vaccine adjuvants monophosphoryl lipid A (MPL) and CpG 1018 are both highly characterized agonists of Toll-like receptors (TLRs) and help to shape the immune response towards a Th1-type quality [10]. In many cases, adjuvant products comprise combinations that may include TLR ligands, saponins, and particulate formulations, such as AS01 in Shingrix and Arexvy (liposomal TLR4 agonist and QS-21), CpG 1018-Alum in Corbevax (TLR9 agonist adsorbed to Alum), and Alhydroxiquim-II in Covaxin (TLR7/8 agonist adsorbed to Alum) [10].

Many unique adjuvants and adjuvant systems exist in preclinical development, as has been the case for many years. However, there continues to be a disconnect between the state of our understanding of adjuvants and our ability to deliver on that understanding in terms of clinical translation. At the same time, it is widely appreciated that adjuvants play a critical role in the development of long-lasting immunity and protection against target pathogens. This is most recently highlighted by the first generation of mRNA vaccines against SARS-CoV-2, which induced impressive initial antibody titers that rapidly waned [15], a liability that adjuvanted vaccine candidates can improve upon [16]. Moreover, much work is needed to establish adjuvant compatibility with next-generation vaccine platforms, including mRNA, DNA, and adeno-associated virus (AAV)-based vaccines. The gap between our understanding of the need for adjuvants and our ability to capitalize on their use in clinical products is related to multiple factors, including a lack of appreciation for the nuanced formulation considerations in vaccine adjuvant development, unclear rationale or parameters to select one adjuvant over another, and a failure to consider adjuvants with product potential until too late in the development process. At the same time, there are avenues for further development and discovery in adjuvant systems that will make them more accessible and simplify their integration into emerging vaccine systems.

**Figure 1:**
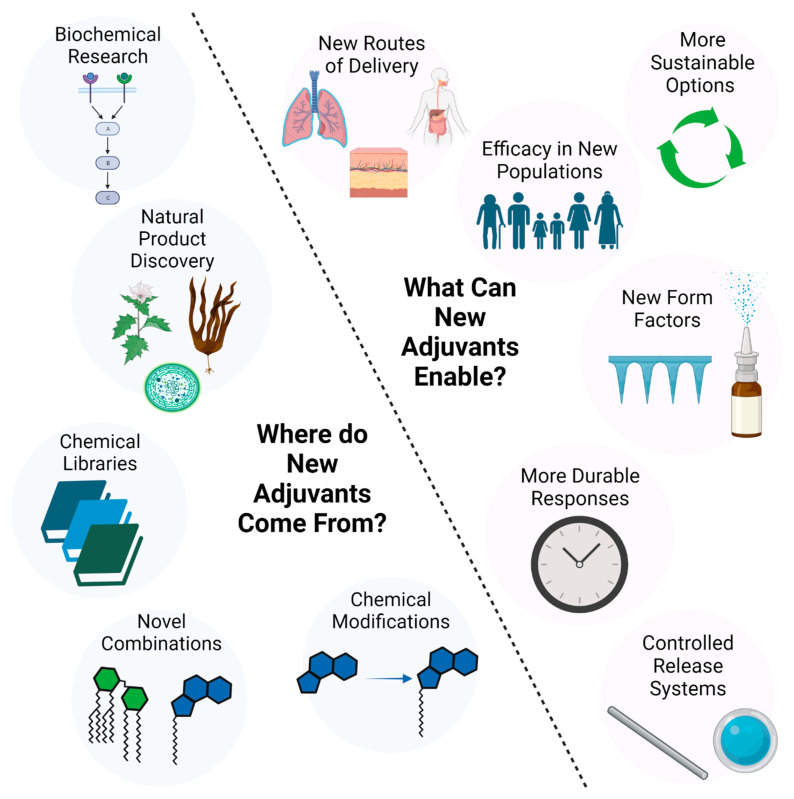
Where do novel adjuvant systems come from, and why are they important? Figure was created with Biorender.com (accessed on 26 June 2023).

From this perspective, we attempt to summarize what steps can be taken and what factors can be considered in the early stage of vaccine adjuvant design and development. We overview the importance of sustainable material sourcing, storage stability, alternate routes of administration, controlled release kinetics, and methods the vaccinologist can use to weigh these options while positioning their product toward clinical translation (Figure 1).

## 2. Sustainable Raw Materials

Natural product discovery generally refers to the practice of identifying compounds in existing natural reservoirs, usually plants, fungi, or bacteria [17,18]. In an idealized process, the specific active compound is identified via fractionation and a high-throughput bioassay [17]. Next, a highly refined purified compound is generated, then physical and analytical chemistry techniques are employed to elucidate its active structure (e.g., nuclear magnetic resonance, time-of-flight mass spectrometry, and Raman spectroscopy). The active compound is synthetically produced via traditional organic chemistry techniques; then, medicinal chemistry approaches can be employed to refine the drug characteristics of the final active pharmaceutical ingredient (API). This approach generally relies on the eventual ability to fully synthesize the compound of interest, as chemical extraction of refined compounds is challenging and costly to scale.

Despite the associated costs and challenges, many existing adjuvants are derived directly from refined natural products. The saponin pool that QS-21 is refined from comes from *Quillaja saponaria*, the soap bark tree, which is native to a relatively small area of central Chile [13]. Saponins from soap bark trees were first studied as immunostimulants in the first half of the 20th century and were later refined into Quil A, a heterogeneous saponin mixture that is presently used as an adjuvant in veterinary vaccines. In an effort to reduce the reactogenicity of Quil A, scientists identified and isolated the particularly active component QS-21 (named as the 21st peak on a chromatogram of Quil A) [13]. The resulting QS-21 is a blend of two isomeric compounds differentiated by a terminal sugar structure [19]. Despite the relatively low-yield industrial process, QS-21 is presently included in several vaccine products, including Shingrix, Mosquirix, and Arexvy, and is one of the multiple saponins in Nuvavoxid. As the number of QS-21-containing vaccines increases, alternative industrial processes to obtain QS-21 or suitable analog molecules could be required due to the limited availability of *Quillaja saponaria* and the poor extraction yields [20,21]. For example, synthetic biology approaches, such as expressing saponins in bioengineered organisms, may provide a more sustainable avenue to saponin production [22]. Synthetic or semi-synthetic approaches to generating QS-21 analogs have also been demonstrated [23].

Squalene provides another example of a natural product with a long history of use as an adjuvant vaccine component. All current squalene-based pharmaceutical products use refined oil derived from shark livers, including those containing MF59 or AS03 [24]. While it is difficult to quantify how an increase in squalene-based vaccine adjuvant demand would impact shark populations, a more sustainable source of squalene or alternative analogs are desirable objectives. In recent years, many cosmetic companies have replaced shark squalene with olive-derived squalene. Olive squalene is concentrated in the skin and seeds of the fruit, meaning that it can be a byproduct of the global olive oil industry. Emulsified olive-derived squalene appears to maintain adjuvant properties in preclinical testing [25,26]. Other companies are working to distill squalene from the amaranth grain, which can be selectively cultivated and processed to increase squalene production [27]. However, due in part to the difficulty and cost associated with extracting highly purified squalene from plants, plant-derived squalene has not yet been employed in any FDA-approved vaccine product [24]. Other plant-derived terpenoid structures have also demonstrated adjuvant properties and merit further investigation [28,29]. Alternative routes to a more sustainable source of squalene-like molecules include synthetic biology and synthetic chemistry approaches. For example, non-shark squalene or squalene analog emulsions obtained using bioengineered yeast and/or synthetic chemistry have demonstrated equivalent or improved adjuvant activity compared to shark squalene emulsions [28,30,31]. These approaches have the advantage of existing infrastructure in microbial fermentation and industrial chemical engineering. An added benefit of generating analog structures is that structure-function relationships can help elucidate features important for improved adjuvant activity in emulsions containing squalene-like molecules [28].

MPL, a TLR4 agonist derived from *Salmonella minnesota*, was the first TLR agonist to be used in approved vaccines. The manufacturing process of MPL results in a heterogeneous mixture of compounds with different numbers of acyl chains, each of which may have different adjuvant properties [32]. In contrast, synthetic TLR4 agonists offer highly pure single constructs with structures optimized for human TLR4 activity. Several synthetic TLR4 ligands as components of vaccine adjuvant formulations are undergoing clinical testing [33]. However, other synthetic TLR ligands have already reached commercial product approval, including CpG 1018 (TLR9) in the hepatitis B vaccine Heplisav-B and, in some geographies, Alhydroxiquim-II (TLR7/8) in the SARS-CoV-2 vaccine Covaxin. In addition to TLR ligands, other synthetic PRR ligands, such as trehalose dibehenate (TDB), a ligand of the C-type lectin receptor Mincle, have advanced to clinical testing [34]. Synthetic ligands for TLRs and other PRRs offer well-defined structures for precisely engineering the adjuvant properties of next-generation vaccines.

The above examples represent a range of adjuvant types and sources but are not intended to be comprehensive. Many other types of molecular and particulate adjuvants are in development. Nevertheless, regardless of the type of adjuvant and whether it is obtained from natural or synthetic sources, raw material sustainability must be a primary consideration.

## 3. Thermostability

Much has been written elsewhere about vaccine cold-chain storage and the need for thermostable vaccine products to meet global vaccine needs [35,36], which is highlighted by the poor stability profile of mRNA-based COVID-19 vaccines that made them challenging to access in many areas [37,38]. Thermostability generally refers to the ability of the product to remain stable at ambient temperatures for sufficient amounts of time. Because of the logistical challenges of maintaining cold-chain transport, as much as 50% of vaccine doses are rendered unusable before administration in many low- and middle-income countries [39]. In particular, more effort is needed in the development of thermostable adjuvant-containing vaccines [39,40]. Generating a thermostabilized product often necessitates reducing the water activity of a product to slow the rate of hydrolysis and other aqueous-phase decomposing processes, which are the major driving forces in antigen and adjuvant degradation [41,42]. This is often accomplished by generating a dried product, generally either through lyophilization or spray drying [42]. However, transforming liquid adjuvant formulations into solid-state formulations with enhanced thermostability is not a trivial endeavor due to the complexities of adjuvant particulate structures. It is, therefore, worthwhile to consider optimizing formulations for enhanced thermostability early in development. The generation of solid-state materials is generally achieved via some form of drying technology, such as lyophilization or spray drying [43,44,45]. Recent progress in applying such techniques to generate thermostable adjuvant formulations has been encouraging. For instance, a thermostable lyophilized presentation of a vaccine composition consisting of a protein antigen and an oil-in-water emulsion containing a synthetic TLR4 ligand was shown to maintain or even improve the safety and immunogenicity profile of the non-thermostable vaccine in a Phase 1 clinical trial of a GLA-SE adjuvanted subunit tuberculosis vaccine [46]. The emerging class of nucleic acid-based adjuvants, such as TLR3 and RIG-I agonists, is likely to benefit greatly from dried preparations due to its relatively poor liquid stability compared to its improved stability in a dried state [47,48]. Alternative strategies to generate solid formulations include entrapment in an implant or microneedle patch, which may in turn provide controlled release benefits [49,50]. In any case, by prioritizing thermostability in new adjuvant-containing vaccines, more global markets may become accessible, which has clear benefits from economic, ethical, and global health perspectives.

## 4. Alternative Routes of Delivery

Mucosal immunology is a somewhat less-explored field than systemic immunity, despite the mucosal route of infection for many infectious diseases. Each major mucosal system, including the urogenital, respiratory, and gastrointestinal tracts, has its own unique combination of defensive structures and effector cell populations. A large body of evidence demonstrates that vaccines delivered mucosally, such as the pulmonary or oral route, lead to immunological outcomes that are unique from systemically delivered products [51,52,53]. Moreover, mucosal immune responses generated by mucosally delivered vaccines may be more effective in preventing infection for some indications [53,54]. Currently, there are some approved vaccines delivered by the oral or nasal route, but these are usually live-attenuated pathogens that do not contain extrinsic adjuvants. SARS-CoV-2 has generated even greater interest in the potential for mucosal delivery of vaccines [55,56]. However, several challenges must be overcome for more widespread adoption of mucosally delivered adjuvant-containing vaccines, including issues associated with absorption, toxicity, and administration technique.

The absorption of intact biomolecules across mucous membranes has been a clinical challenge for decades. Compared to parenterally dosed vaccine products that offer immediate bioavailability, only a fraction of mucosally administered material may successfully pass through epithelial tissue to generate a productive interaction with the effector cell populations residing below. This is especially true for mucosally delivered biologics, such as protein antigens or nucleic acids, which often have single-digit bioavailability percentages, even in advantageous mouse models [57,58,59]. This low permeability stems from the relative lack of diffusion of large biomolecules across intact epithelial tissue, in addition to ubiquitous chemical and enzymatic proteolysis. In this regard, pulmonary dosage forms may behave more favorably due to a large surface area, a high degree of vascularization, and relatively thin mucosal barriers [60]. In any case, mucosal adjuvant formulation development efforts may be needed to target cell uptake by resident antigen-presenting cells, which can sample antigens from across the epithelial barrier and then present them to effector subsets in the draining lymph node. Optimization of particle size and material properties are often exploited to achieve the desired tissue deposition [61,62].

Due to the issue of potential toxicity of mucosally delivered vaccine adjuvants, additional considerations must be taken into account compared to parenteral delivery. Most adjuvants generate some kind of local inflammation, which, depending on the individual and the product, could result in some short-term local or systemic reactogenicity [63]. This disruption to tissue homeostasis is more pronounced in mucosal tissues, such as the lung or nose, where there might be more acutely undesirable side effects due to epithelial tissue inflammation. For this reason, the inclusion of adjuvants in mucosal vaccines is challenging because, while there is a need to elicit potent localized immune responses, there is also a more overt risk to doing so. The widely publicized experience with a nasally delivered inactivated influenza vaccine containing a bacterial toxoid adjuvant that caused Bell’s palsy in some recipients provides an unfortunate warning lesson in this regard [64].

Finally, mucosally delivered vaccine and adjuvant preparations need to take the administration technique/device into account. Because of their limited application in current clinical products, new vaccine dosing methods will pose an adoption challenge. Intramuscular injections are a well-defined and ubiquitous means of administering vaccines, which offers clarity to both the patient and provider that the vaccine was administered successfully. In contrast, correct and incorrect user techniques may occur with equal frequency in nasal or pulmonary delivery [65]. The absorption of oral products is similarly challenging and may be affected by a host of factors, including an individual’s diet before/after vaccination, microbiome status, enteropathy, stress, or presence of enteric pathogens or other conditions, such as inflammatory bowel disease [66,67]. In any case, it is essential that device compatibility with the adjuvant formulation is comprehensively assessed so that optimal delivery performance can be achieved [62].

In summary, emerging adjuvant systems hoping to capitalize on the benefits of mucosal administration need to be evaluated thoroughly by taking the above factors into account. Thus, it is unlikely that an adjuvant formulation that has been designed for intramuscular use will automatically be optimized for mucosal delivery without some modifications to the composition. Furthermore, there must be a clear benefit demonstrated compared to more traditional delivery approaches that justifies the need for investing in mucosal delivery development efforts. For example, for vaccines against highly contagious respiratory pathogens, such as SARS-CoV-2 or influenza, an obvious added value would be if an adjuvanted vaccine could induce sterilizing immunity at the site of infection rather than being limited to primarily reducing disease severity.

## 5. Control of Exposure Duration and Kinetics

In recent years, a number of reports have looked at the use of implantable controlled-release systems as a means to control the kinetics of vaccine antigen and adjuvant exposure. In general, extended, sustained, or pulsatile exposure to vaccine components over the course of weeks to months can improve the potency of an immune response and support the development of a broadly neutralizing antibody response [68,69]. This is especially relevant when developing vaccines against highly challenging pathogens, such as HIV [70,71]. These implantable systems also have the potential to enable single-intervention vaccine regimens, eliminating the need for booster doses [72]. There has also been extensive preclinical research into the use of intertumoral implantation of extended delivery systems for immune-oncology agents, with and without surgical resection, as a means to improve the efficacy of cancer immunotherapies [73]. While our mechanistic understanding of these controlled-release systems is still evolving, it is generally thought that prolonged exposure to an antigen at draining lymph nodes leads to the development of more mature T and B effector cell responses [74].

Controlled-release systems are generally designed for use with protein subunit antigens, and many have incorporated adjuvants to enhance the immunogenicity of these vaccines. To this end, many adjuvants have been included in a number of controlled-release vaccine systems, but most are off-the-shelf materials as opposed to fit-for-purpose compositions, including MPL, Alum, and CpG (among others) [75]. These adjuvants, while effective, are unlikely to be optimal in the unique kinetics of a controlled-release system without additional modification. It is, therefore, worthwhile to test adjuvant platforms in the context of extended-release systems now so that, once the technology reaches clinical maturity, there will be front-runner adjuvants that could be applied in a second-generation product.

From a design perspective, implantable vaccines that have an active lifespan of weeks to months would be best served by a biodegradable material; otherwise, the requirement for removal of the device defeats many potential patient benefits. To this end, many biodegradable materials have been explored, including poly(D,L-lactic acid-co-glycolic acid) and polyanhydride as implants and various materials in gels, such as a variety of polyester and polyethylene block-copolymers, polysaccharide polymers like alginate or chitosan, synthetic peptide scaffolds, and cellulose derivatives (among others) [75]. From a use standpoint, vaccine products, in general, are dependent on high uptake in relevant populations, meaning that dosing procedures that are more complex than a standard percutaneous injection will be more challenging to market [76,77]. For this reason, shear-thinning or in situ crosslinking materials are likely to have a market advantage over solid form factors that will require more complex outpatient procedures to place correctly [75]. A potential advantage of these novel polymer-gel systems is that adjuvants can interact directly with the chemistry of the material to better synchronize release with a protein antigen [78,79]. Another approach is to use aluminum oxyhydroxide as a carrier for both the protein antigen and an anionic adjuvant, linking the release of both components [72,80]. Lastly, the largest hurdle adjuvant-containing systems will face is the regulatory challenge posed by establishing safety profiles for long-term exposure to inflammatory compounds in healthy populations. Extended exposure to adjuvants is likely to be a concern for both regulatory bodies and patients, so a very high bar for safety must be achieved in preclinical and early clinical studies. Indeed, long-term exposure to even sub-therapeutic amounts of pro-inflammatory compounds might have unintended risks [68,69]. These concerns are of course different in the field of oncology, where sustained systemic exposure to one or even multiple pro-inflammatory immune oncology drugs is becoming a standard of care for many indications [81,82]. So, there is a reason to anticipate that a tumor-targeting vaccine implant may be the first to market, with subsequent prophylactic vaccination efforts for HIV or other difficult-to-target pathogens following.

## 6. Combining Multiple Adjuvants and Vaccine Platforms

The success of the AS01 adjuvant system has demonstrated the potential clinical value to be realized with combinations of immunostimulants. AS01 contains the TLR4 agonist MPL and the fractionated saponin QS-21, formulated in a liposome that is admixed with or used to reconstitute the vaccine antigen [83]. Studies have shown that AS01 benefits from an inherent synergy between the two agonists, leading to enhancements in both cellular and humoral immunity [84,85]. Interestingly, AS01 has often outperformed AS02 in clinical testing, notably in the development process of the now WHO-prequalified malaria vaccine Mosquirix, even though AS02 also contains MPL and QS-21 but is formulated in an oil-in-water emulsion [86]. Thus, it is critical to consider not only the activity of each immunostimulant separately but also how they function together, how best to formulate them, and how the adjuvant formulation interacts with the vaccine antigen. Regardless of the actual components, the goal of adjuvant systems that combine multiple immunostimulants is to generate a complementary immune response without approaching the reactogenicity that might arise from using higher doses of a single immunostimulant. This requires identifying a synergistic effect between two agonists, which is most likely to occur between agonists that act in at least a partially orthogonal manner or target different cell types, as opposed to the additive effect that would be observed between two agonists that act along identical pathways. For instance, MPL and QS-21 are thought to act through complementary immunomodulatory pathways, resulting in a synergistic combination. Many other multi-agonist combinations have been trialed in preclinical models, notably the nucleic acid-based adjuvants CpG and 2′3′-cGAMP and the TLR4 agonist GLA with the TLR7/8 agonist 3M-052 [87,88]. Formulations of particulate adjuvants (such as Alum or squalene emulsion) with TLR ligands should also be considered combination adjuvants, as well as delivery vehicles for adsorbed agonists and/or antigens (e.g., AS04 and GLA-SE). Alum, in particular, is effective at binding protein antigens and molecular agonists and facilitates the codelivery of both to relevant immune populations in the draining lymph node, such as in the HPV vaccine Cervarix [89].

Until recently, any given infectious disease had only a few commercial vaccines, and at any given time, most of the available vaccines for that disease used similar modalities (live attenuated, whole killed virus, subunit, virus-like particle, etc.). In the wake of the 2019 coronavirus pandemic, where nucleic acid and adenoviral vector vaccines both reached global markets alongside more traditional protein subunit or inactivated virus vaccines, there were expanded opportunities to consider how different vaccine modalities may interact with each other. Similar to how components of combination adjuvants, such as AS01, have an innate synergy, leading to more effective immune responses, it is reasonable that different vaccine modalities might also demonstrate beneficial complementarity. These kinds of vaccine schedules, where an individual receives different products between the prime and subsequent boost immunizations, are generally referred to as heterologous combinations [90,91]. The data obtained from heterologous COVID-19 vaccine regimens have generally focused on the near-term immunogenic profile, where the highly potent mRNA vaccines appear to be the top performers. However, it has become clear, in the context of SARS-CoV-2, that long-lasting protective immune memory has been more elusive to achieve with the current vaccines, particularly in the presence of rapidly mutating virus strains [92]. In some cases, other vaccine platforms, such as appropriately adjuvanted subunit proteins or nanoparticle presentations, may provide distinct advantages, such as increased durability or breadth [16,93,94]. By presenting a combination of antigen types and structures in different vaccine platforms, including adjuvants where appropriate, it may be possible to better shape the quality of the final immune response [95]. Even simultaneous administration of multiple vaccine modalities may be worth considering [96]. However, the efficacy of mRNA-based vaccines may be suppressed when dosed along potent activators of type-one interferons (e.g., TLR3, TLR7/8, TLR9, STING, and RIG-I agonists) due to a reduction in protein expression [97,98,99]. Nevertheless, there is potential that orthogonal immune potentiators might improve the limited durability of existing mRNA vaccines. To this end, an increased understanding of the interaction between different adjuvants, vaccine modalities, and routes of delivery might improve our ability to respond to pandemics.

Another approach to heterologous vaccination is alternating the route of administration to generate more effective mucosal immunity. This kind of “prime and pull” strategy was first described as a means to improve T cell responses in the lower female genital tract to a herpes simplex virus 2 (HSV2) subunit vaccine [100]. The theory is that by first vaccinating via a conventional route (i.e., intramuscular) to elicit a mature systemic T cell response, peripheral T cell populations can then be recruited to mucosal sites using the local delivery of chemokines or other adjuvants. This approach has recently been applied as a therapeutic vaccine candidate in an animal model of HSV2 and demonstrated that the prime and pull strategy was more effective than either the systemic or mucosal approach in isolation [101]. In addition to providing protection in the urogenital tract, prime and pull strategies have been effective in animal models of nasal, pulmonary, and ocular infection [102,103,104,105].

**Figure 2:**
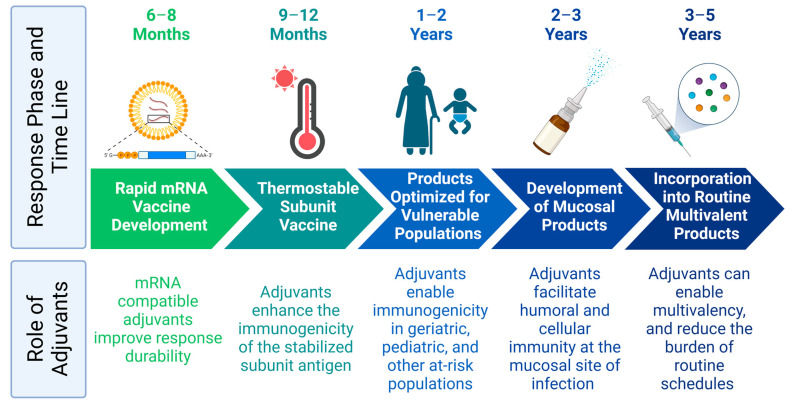
Careful selection of adjuvants streamlines future pandemic responses. Figure was created with Biorender.com (accessed on 26 June 2023).

The necessary collaborations between vaccine manufacturers required to clinically evaluate these combinations could present substantial complexities; however, similar cross-branded combinations are commonly employed in oncology and are often beneficial to both manufacturers and patients [106]. Given that some technologies will be first to market, it could be advantageous to design later products to provide complementary boost responses to the initial products. On the other hand, there is a risk that heterologous combinations might be ineffectual. In the context of the COVID-19 pandemic, it was shown in a small clinical cohort that individuals who received the BNT162b2 mRNA vaccine followed by the Novavax subunit boost generated a less substantial immune response than patients who received the BNT162b2 vaccine as a prime and boost. However, patients who first received the Oxford adenovirus vaccine, followed by the Novavax vaccine, outperformed groups who only received the BNT162b2 and/or Moderna mRNA vaccines in terms of T cell activity [91]. This suggests that for difficult indications, considering heterologous combination products earlier on in vaccine development would allow for optimizing each individual product to achieve a subset of clinical endpoints rather than expecting comprehensive immune protection from each product on its own. In the context of pandemic preparedness, this approach might mean that early-stage products are focused on generating strong systemic humoral immunity to reduce disease severity, whereas subsequent products can be designed to emphasize enhanced durability or mucosal responses (Figure 2).

## 7. Optimization via Aggregate Metrics

The increased efficacy of AS01-based adjuvants compared to AS02 is somewhat surprising considering the well-accepted adjuvant properties of squalene emulsions, even without the addition of receptor agonists, and the generally immune inert nature of conventional liposomes [86,107,108]. This suggests that the immune response generated from multiple immune stimulants may not be well modeled by a simple linear combination of the inputs. In practice, the mechanistic interplay between different immunostimulants is often challenging to define fully, and empirical studies remain the typical approach for adjuvant selection. However, characterizing immune responses to a combination of immunostimulants might be better achieved using tools and models built for complex physical systems, such as those found in digital signal processing or microscopy, where the specific underlying features that dictate the change from a signal input to output are governed by an empirical state-space model [109]. State-space models and similar control theory techniques, such as transfer function analysis, are used across multiple engineering fields in the development of new processes or products as a way to predict performance during the design process [110,111]. Alternatively, these models can be derived empirically from systems using specific test signals [112]. This approach is most useful when systems can be approximated as linear time-invariant (LTI) systems, meaning that multiple inputs can be condensed into a linear combination of inputs and that the system state does not vary as a function of time [113]. In the case of immune responses to adjuvants, the immune system is known to be highly non-linear, as exemplified by synergies and anti-synergies between different immunostimulants, and immune responses are highly dynamic and time-variant, as exemplified by immune memory as well as T cell exhaustion and other metabolic phenomena. These complications mean that modeling of the immune system is unlikely to completely replace empirical adjuvant screening; however, techniques such as systems immunology and statistical design of experiments (DoE) might improve the efficiency of the adjuvant selection process.

Systems immunology, the study of immune interactions on a pathway and cell/tissue level in response to different stimuli, has been a remarkable tool to discern how signals are propagated through the immune system and uncover unknown mechanisms of action for immune agonists [114,115]. Like other clinical systems biology approaches, systems immunology seeks to maximize the amount of data that can be generated from minimal biological samples, most often peripheral blood. Techniques vary depending on the goals of the study but often include mass cytometry, single-cell transcriptomics and proteomics, bead-based analysis of soluble factors (i.e., Luminex assays), and more conventional readouts, such as antibody titer and breadth. These high-dimensional datasets are then analyzed using bioinformatics techniques and machine learning algorithms to build models that enable hypothesis testing and forecasting [115,116]. Systems immunology has provided insights on a range of products, from predicting clinical responses to the yellow fever vaccine to clarifying cellular responses to the BNT162b2 mRNA vaccine [117,118]. For example, the B cell responses to the yellow fever vaccine can be correlated almost 1:1 to the expression of the TNFRS17 B cell growth factor receptor, which can be measured as soon as 7 days after vaccination as a more accelerated means of determining successful vaccination than traditional antibody titers [117]. Systems analysis has also been applied clinically to better understand the mechanism of action and underlying kinetic differences between seasonal influenza vaccines with or without the addition of MF59 [119]. These approaches provide an unprecedented ability to characterize immune responses and even confirm the immunogenicity of vaccines weeks to months before more conventional methods, such as antibody titer. Such systems vaccinology approaches will likely have an increasing impact on assessing immune response profiles in clinical studies of experimental vaccines and adjuvants [1,120].

Because of the large amount of data output that can be measured via systems immunology methods, and the large number of input factors that can be considered in adjuvant design and dosing, techniques are needed that can simultaneously account for and optimize multiple parameters at once. These techniques, especially ones that would be useful in early measurements of human immune responses (i.e., a Phase 0 study), could derisk future clinical studies [121]. A method commonly employed by formulation scientists and engineers is the DoE approach [122,123]. DoE is a method to optimize experimental efficiency and multiple input parameters simultaneously. By applying a subset of the input space, researchers can use their empirical results to extrapolate toward the larger output space, often referred to as a response surface [124,125]. Optimized combinations of inputs are determined by applying desirability weights to each output and running an optimization algorithm on the resulting response surface to identify a set of inputs that will yield a desired set of outputs. DoE is a prospective method, meaning that the experimental groups are designed via a statistical tool set before the study is carried out to minimize the total number of replicates and groups needed to capture the system response to the input parameters to the desired degree. Once the inputs and outputs are programed into the statistical software, a set of experimental conditions and sample sizes are generated that will enable a model of each output as a function of each input and potentially higher order combinations of inputs as a measurement of interactions [122]. Depending on the needs of the researcher, this can be used to determine which input factors have an effect on the desired output and which input variables can be simplified in future studies [126]. Alternatively, once the input set is refined, the output models can be used to identify the optimal conditions of each relevant input parameter. Because of the group-reduction nature of DoE, the optimal combination of input parameters might not be a group that was physically tested but rather inferred from the model of the system [88,127].

In the context of preclinical adjuvant development, DoE inputs could include the dose of one or more PRR agonists, the composition of formulation active ingredients and excipients (such as Alum, squalene emulsion, or liposomes), the frequency of booster administration, the route of delivery, etc. The outputs for this kind of system could be maximizing serum antibodies and cytokines, peripheral cellular activation via flow cytometric measurements, measurements derived from tissues sampled at the conclusion of the study (i.e., long-lived antibody-secreting cells in bone marrow, splenocyte/lymph node activation, mucosal antibodies), neutralizing antibody titers, upregulation of genes in systems immunology datasets, or even physicochemical stability of the adjuvant formulation. Other outputs could include minimizing reactogenicity or undesirable immune response signals (e.g., Th2 vs. Th1 cellular immunity). The outputs can be integrated into a single score for ranking various candidates using an aggregate tool such as the desirability index approach [88]. A number of groups have demonstrated desirability index or DoE-based optimization in the development of preclinical and even clinical vaccine products, including the optimization of the ratio of 3M-052 and GLA in a preclinical *Entamoeba histolytica* vaccine candidate or the optimization of the excipient content and stability of a lyophilized subunit tuberculosis antigen with GLA-SE adjuvant that has now been validated in a Phase 1 clinical trial [46,88,126,127,128,129,130]. Applying the high-dimensional data that can be generated using systems vaccinology approaches together with the efficient multi-parameter optimization of DoE techniques enables extracting as much information as possible while minimizing the size of groups required for testing.

## 8. Conclusions

Considering that many vaccines are well served by existing adjuvants, what benefit could new vaccine adjuvants address that is not currently met by existing products? One important practical point is access to the adjuvant. In many cases, it may not be possible to access existing adjuvants due to licensing restrictions, limited supply, or related business decisions. Additionally, new, rationally designed adjuvants might lead to more desirable immune response qualities or better meet the needs of specific populations or geographies. In any case, it is essential that the added value of any new adjuvant system be clearly benchmarked to well-accepted, widely available adjuvants, such as Alum or squalene emulsion [131]. Another practical factor that should be considered early in the development of new adjuvant systems is the balance of simplicity and innovation. In general, most pharmaceutical systems benefit from being as simplified as possible, from formulation and composition to manufacturing and distribution. A simpler product that contains fewer ingredients, and is manufactured in fewer steps, has fewer potential points of failure, fewer risks of supply chain disruption, reduced regulatory burden, and a lower production cost. However, there are obvious benefits to mindfully accepting some additional risk or complication in order to generate a more effective product. For example, over the last 40 years, numerous HIV vaccine candidates based on standard approaches have failed [132], so there is a clear need to innovate to develop new vaccine and adjuvant technologies to address these challenges. However, such innovations must still be globally accessible in terms of material availability, vaccine stability, dosing strategy, and scale of manufacturing. These factors need to be considered early on in development to ensure that clinical translation is feasible (see Box 1).

Although vaccines containing novel adjuvants have historically been one of the most challenging products to bring to the clinic, multiple adjuvant-containing vaccines have been approved in the last ~15 years, with SARS-CoV-2 substantially accelerating this trend. Furthermore, initial clinical progress has been achieved in the development of thermostable vaccine adjuvant platforms as well as the implementation of systems immunology approaches to analyze clinical samples. In parallel to these clinical advancements, significant preclinical progress has been made regarding sustainable sourcing of adjuvant raw materials, alternative routes of adjuvant delivery, and control of vaccine and adjuvant delivery kinetics, providing new opportunities to optimize vaccine adjuvant design, development, and manufacturing. In general, vaccine adjuvant development is a highly contextual endeavor, and there is no one-size-fits-all solution. However, there is hope that progress in vaccine adjuvant development and accessibility can be accelerated further by taking into account the practical aspects of adjuvant development discussed here.

Box 1 **Summary of recommendations for new adjuvant development.**

1. Derisk the environmental and supply chain sustainability of materials. Consider alternate compounds or production methods to reduce dependency on animal or unsustainable sources.
2. Incorporate thermostability approaches early in development.
3. Define the need that a new adjuvant would address (e.g., mucosal immunity and durability of immunity) and tailor subsequent activities to this need.
4. Keep compositions as simple as possible but consider the added value of combination approaches when necessary.
5. Employ experimental design methods and systems immunology approaches to comprehensively characterize the immune profile of the new adjuvant.

## Data Availability

No new data were created or analyzed in this study. Data sharing is not applicable to this article.
